# Annual trends of ophthalmic surgeries in Japan’s super-aged society, 2014–2020: a national claims database study

**DOI:** 10.1038/s41598-023-49705-x

**Published:** 2023-12-18

**Authors:** Saori Wada, Masahiro Miyake, Masayuki Hata, Ai Kido, Takuro Kamei, Masahiro Akada, Shusuke Hiragi, Hiroshi Tamura, Akitaka Tsujikawa

**Affiliations:** 1 Department of Ophthalmology and Visual Sciences, Kyoto University Graduate School of Medicine, 54 Shogoin, Kawahara, Sakyo, Kyoto 606-8507 Japan; 2 Kyoto Okamoto Memorial Hospital, Kyoto, Japan; 3 Medical Research Institute KITANO HOSPITAL, PIIF Tazuke-Kofukai, Osaka, Japan; 4 Center for Innovative Research and Education in Data Science, Institute for Liberal Arts and Sciences, Kyoto University, Kyoto, Japan

**Keywords:** Glaucoma, Lens diseases, Retinal diseases, Epidemiology

## Abstract

This study aimed to analyze the trends and factors influencing the number of ophthalmic surgeries in Japan using the open data from the National Database of Health Insurance Claims and Specific Health Checkups of Japan published by the Ministry of Health, Labour and Welfare. We calculated the number of cataract, glaucoma, and vitreoretinal surgeries, categorized by sex, age, and surgical type, for the fiscal years (FY) 2014 to 2020. The number of cataract surgeries remained stable at approximately 1.45 million cases from FY 2014 to 2018, increased to nearly 1.6 million cases in FY 2019, and decreased to 1.45 million cases in FY 2020. Among glaucoma surgeries, surgical treatments were increased 1.8 times over 7 years, from 33,000 to 60,000 cases. Laser treatment remained steady at around 55,000 cases from FY 2014 to 2017 and then increased to approximately 60,000 cases. The number of vitreoretinal surgeries was increased 1.2 times from FY 2014 to 2019, from 120,000 to 140,000, and decreased to 130,000 by FY 2020. Trends in ophthalmic surgeries over the past 7 years may be influenced by population aging, minimally invasive surgery, and the coronavirus disease pandemic. These findings have implications on surgical decision-making and resource allocation.

## Introduction

Understanding the trends in the number of ophthalmic surgeries is important because it has significant implications for healthcare planning and resource allocation. A key factor driving the need for ophthalmic surgery is the aging populations in developed countries. In many developed countries, especially Japan, while the total population has been declining, there has been a steady increase in the proportion of older individuals aged 65 years and above^1^. As of 2020, Japan is the only “super-aged country”, that is the country whose proportion of older individuals aged 65 years surpasses 20%, according to the definition by the United Nations, while the number of super-aged countries will rise to 11 in 2050^2^. Because this demographic shift is particularly relevant to ophthalmology, as age-related eye conditions such as cataracts and glaucoma become more prevalent among the older population, examining the trends in ophthalmic surgery in Japan is crucial for forecasting global trends in the number of ophthalmic surgeries in the future.

An increase in the older population is expected to increase the demand for cataract and glaucoma surgeries. Cataracts are common age-related conditions that cause significant visual impairment and may require surgical intervention to restore vision. Similarly, glaucoma, a progressive eye disease often associated with increased intraocular pressure, can lead to irreversible vision loss if not treated in a timely manner. Given the increasing prevalence of these conditions in the older population, an increase in the number of surgeries performed to address these issues is also expected.

In recent years, there has been growing interest in minimally invasive surgical techniques in the field of ophthalmology. Procedures, such as micro-invasive glaucoma surgery (MIGS) and micro-invasive vitrectomy surgery (MIVS), have gained popularity due to their potential benefits, including reduced complications, shorter recovery times, and improved patient outcomes. These approaches provide alternative options to traditional methods and have expanded surgical indications. Consequently, the number of minimally invasive ophthalmic surgeries is increasing as surgeons gain proficiency and enhance safety profiles.

In this study, we aimed to investigate trends in the number of ophthalmologic surgeries in Japan. To this end, we utilized the National Database of Health Insurance Claims and Specific Health Checkups of Japan (NDB). This comprehensive national claims database, administered by the Ministry of Health, Labour and Welfare (MHLW), covers the entire Japanese population of approximately 126 million individuals and captures almost all medical treatments received, including data from both inpatient and outpatient^3–9^. The advantage of using NDB data lies in its high comprehensiveness and scale. These data provide valuable resources for examining patterns and changes in healthcare utilization. Our previous reports using NDB focused on the epidemiology of various ophthalmic diseases^3–6^. This study aimed to gain insights into the trends and factors influencing the number of ophthalmic surgeries in Japan by leveraging the NDB open data, which includes fundamental tables summarizing surgeries based on various factors.

## Methods

This was a retrospective and descriptive study using NDB open data published and managed by the Japanese MHLW. All investigations adhered to the principles of the Declaration of Helsinki. Ethical review was exempt because we used a public database (https://www.mhlw.go.jp/stf/seisakunitsuite/bunya/0000177182.html) for analysis, and the data contained no identifiable personal information.

### Database

Detailed information on the NDB has been described in our previous studies^3–6^. The universal health insurance system in Japan covers the entire population of 126 million individuals. All claims data were principally submitted electronically and stored in the NDB, which has been managed by the MHLW since 2011^3,4,7–9^. The NDB contains information on medical claims in Japan, including diagnoses coded according to the *International Statistical Classification of Diseases and Related Health Problems, Tenth Revision* (ICD-10), drugs, and procedures for both outpatients and inpatients.

The MHLW has created basic summary tables by statistically aggregating NDB data along various axes, which are published as NDB open data on the web^9,10^. The first NDB open data were published in October 2016 and summarized the claims data for the fiscal year (FY) 2014. Currently, up to the 7th NDB open data have been released (as of April 22, 2023). We were able to access the first to the 7th NDB open datasets, which covered the period from FY 2014 to FY 2020.

Age is used as a categorical variable in the NDB open data. If one category had count data of less than 10, all data of the strata were undisclosed according to the MHLW regulations^10^. Thus, age-stratified or age and sex-stratified data were missing for some procedures.

### Data analysis

Each surgical procedure is assigned a local code in Japan. Additionally, according to the complexity of the surgery, surgical codes are subclassified into medical practice codes. Online Supplementary Table 1 shows the correspondence table between the codes (surgical and medical practice codes) and surgeries (cataract surgery, glaucoma surgery, and vitreoretinal surgery). Cataract surgery was defined according to the surgical codes K282 or A400. Glaucoma surgery was defined using the surgical codes K268, K270, K271, K272, or K273, which were sub-classified into surgical treatments (K268, K272) and laser treatments (K270, K271, K273). Surgical treatments included peripheral iridectomy, trabeculotomy, trabeculectomy, glaucoma drainage device without a plate (GDD [p−]), glaucoma drainage device with a plate (GDD [p+]), trabecular micro-bypass stent with phacoemulsification (iStent), and cyclocryotherapy. In Japanese medical fee claims, GDD (p−) means the EX-PRESS glaucoma filtration device and GDD (p+) means tube shunts, such as the Ahmed glaucoma valve and Baerveldt glaucoma implant. Laser treatments include laser iridectomy, laser cyclophotocoagulation, and gonio-photocoagulation. Vitreoretinal surgery was defined using the surgical codes K275, K280, K280–2, K281, or K281–2. Vitreoretinal surgery was subdivided into vitrectomy (K280, K280–2, K281, and K281–2) and scleral buckling (K275). Vitrectomy includes pars plana vitrectomy, intraocular endoscopic surgery, proliferative vitreoretinopathy surgery, and retinal reconstruction. The numbers of cataract, glaucoma, and retinal vitreous surgeries for each FY were aggregated by age group, sex, and surgical type. The mean age was calculated using a previously reported method^11^. To calculate the mean using a categorical variable, the median value of each categorical interval was used. The frequencies of each category were multiplied by its median value, and these values were summed and divided by the total frequencies.

## Results

The characteristics of patients who underwent surgery in FY 2014 and FY 2020 are shown in Table 1. The total number of cataract and glaucoma surgeries was higher in females than in males in both FYs, and the total number of vitreoretinal surgeries was higher in males than in females. Overall, females who underwent surgery were older than males who underwent surgery in both FYs. In all surgeries except for scleral buckling, the mean age of the patients was > 60 years, while that of patients with scleral buckling was approximately 40 years in both FYs.

**Table 1:** The characteristics of patients in the fiscal year 2014 and 2020.

Surgical type	Fiscal year 2014						Fiscal year 2020					
	Number of surgeries			Age (year; mean)			Number of surgeries			Age (year; mean)		
	Total	Male	Female	Total	Male	Female	Total	Male	Female	Total	Male	Female
Cataract surgery	1,415,267	610,447	804,540	74.1	73.2	74.7	1,448,997	657,155	791,698	74.1	73.4	74.7
Glaucoma surgery	88,019	33,757	56,463	71.3	69.6	72.3	120,655	51,135	68,751	71.8	70.8	72.5
Surgical treatment	33,340	16,864	16,244	69.5	67.8	71.2	60,108	29,974	29,596	71.9	70.8	73.0
Peripheral iridectomy	1608	429	1127	73.4	71.7	74.0	1209	387	705	73.7	73.4	73.9
Trabeculotomy	10,957	5036	5921	69.6	67.7	71.3	28,900	13,670	15,163	72.4	71.3	73.4
Trabeculectomy	14,644	7933	6680	68.7	67.3	70.3	16,582	9113	7419	69.6	69.0	70.9
Tube shunt implantation without plate (GDD [p−])	5200	2929	2243	71.0	69.6	72.8	3789	1797	1477	63.2	71.8	74.7
Tube shunt implantation with plate (GDD [p+])	817	512	263	63.9	62.0	67.7	2773	1850	1145	74.7	67.2	72.4
Trabecular micro-bypass stent with phacoemulsification	–	–	–	–	–	–	6859	3157	3677	75.0	74.7	75.2
Cyclocryotherapy	114	25	10	72.8	66.9	87.5	102	–	10	82.5	–	82.5
Laser treatment	54,679	15,440	39,149	72.5	71.8	72.7	60,547	21,161	39,155	71.7	71.0	72.2
Laser iridectomy	43,518	10,106	33,389	72.5	72.3	72.6	33,661	8592	25,025	71.9	71.6	72.0
Laser cyclophotocoagulation	517	294	186	68.3	66.0	71.9	2983	1694	1149	71.8	70.5	73.8
Gonio photocpaglation	10,644	5040	5574	72.6	71.3	73.8	23,903	10,875	12,981	71.5	70.5	72.3
Vitreoretinal surgery	120,530	67,123	53,205	64.6	62.8	66.8	132,879	75,147	57,442	65.8	64.5	67.9
Vitrectomy	114,061	62,913	50,966	65.8	64.2	67.8	128,904	72,643	56,027	66.7	65.4	68.6
Pars plana vitrectomy	104,618	56,875	47,701	66.5	65.0	68.2	121,301	67,727	53,489	67.3	66.0	69.0
Proliferative vitreoretinopathy surgery	9166	5943	3211	58.4	56.7	61.5	7465	4906	2520	58.8	57.5	61.4
Intraocular endoscopic surgery	160	60	44	76.2	75.3	77.4	47	–	–	–	–	–
Retinal reconstruction	117	35	10	2.5	2.5	2.5	91	10	18	2.5	2.5	2.5
Scleral buckling	6469	4210	2239	42.7	42.2	43.7	3975	2504	1415	38.2	38.9	38.5

*MHLW prohibits the publication of data which is less than 10 within each group in NDB utilization studies to protect personal information. If the number of unreleased data can be identified by reverse calculation from the total number, all stratified data is treated as undisclosed. As a result, the total number of surgeries does not equal the sum of surgeries on males and females.

**Trabecular micro-bypass stent with phacoemulsification was included in the list of surgeries covered by Japanese National Health Insurance System in 2018.

**Figure 1:**
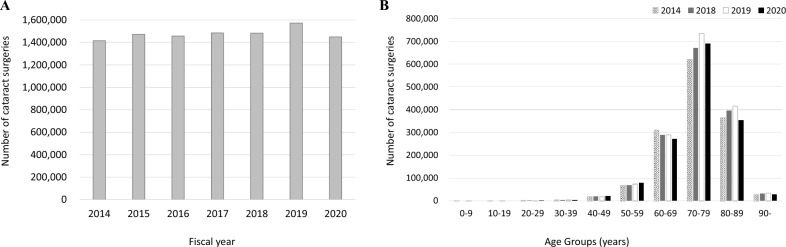
Trend of cataract surgeries. The total number of cataract surgeries (**A**) and age-stratified surgeries (**B**) from the fiscal year (FY) 2014 to 2020 are presented. For age-stratified data, data from FY 2015 to 2017 are missing due to the regulations of the Ministry of Health, Labour and Welfare. While the total number of cataract surgeries remains at approximately 1.45 million cases from FY 2014 to 2018, it increases to nearly 1.6 million cases in FY 2019. Then, it decreases to 1.45 million cases in FY 2020 when the COVID-19 pandemic hit Japan. Focusing on age-stratified data, from FY2014 to 2019, the number of surgeries remains constant for up to 70 years and increased after 70 years. In FY 2020, the number of surgeries performed in patients aged over 60 years decreases.

Figure 1 shows the trend of cataract surgeries; Fig. 1A shows the total number of cataract surgeries from FY 2014 to 2020, while Fig. 1B shows age-stratified surgeries. For age-stratified data, data from FY 2015 to 2017 were missing because of MHLW regulations. While the total number of cataract surgeries remained at approximately 1.45 million cases from FY 2014 to 2018, it was increased to nearly 1.6 million cases in FY 2019; and then decreased to 1.45 million cases in FY 2020 when the coronavirus disease (COVID-19) pandemic hit Japan (detailed numbers for each count are shown in Online Supplementary Table 2). Focusing on age-stratified data, from FY 2014 to 2019, the number of surgeries remained constant for up to 70 years and then it was increased. In FY 2020, the number of surgeries performed in patients aged over 60 years was decreased (detailed numbers for each count are shown in Online Supplementary Table 3).

**Figure 2:**
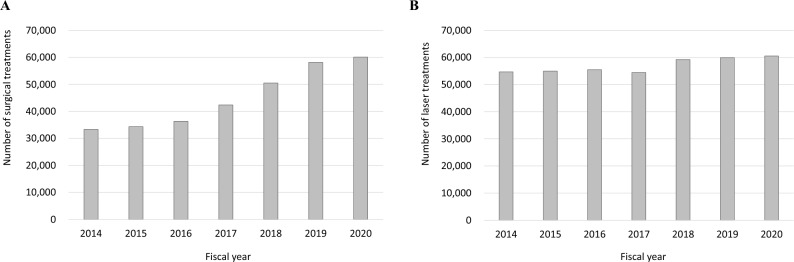
Trend of glaucoma surgeries. The number of surgical treatments (**A**) and laser treatments (**B**) from fiscal year (FY) 2014 to 2020 are presented. Surgical treatments increase 1.8 times for 7 years. Laser treatments remain at approximately 55,000 cases from FY 2014 to 2017 and increase to approximately 60,000 cases.

**Figure 3:**
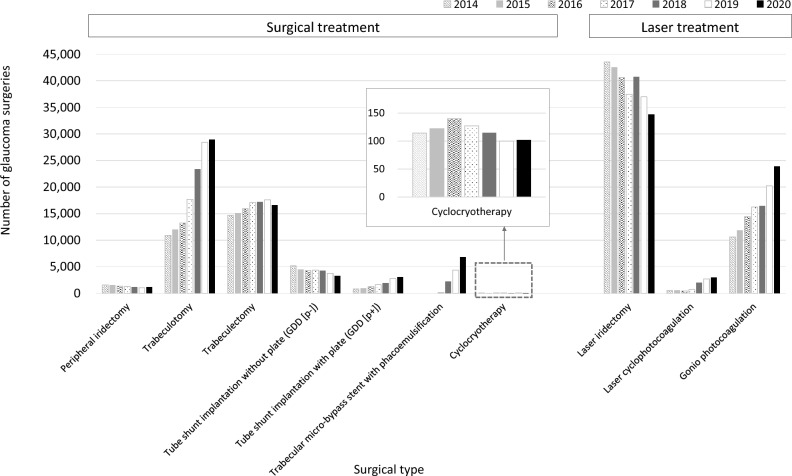
Number of glaucoma surgeries by surgical type. Among surgical treatments, trabeculotomy is the most common in fiscal year (FY) 2020, which accounted for nearly half of all surgical treatments, and was 2.6 times higher than that in FY 2014. Despite an overall increase in surgical treatments, glaucoma drainage devices without a plate (GDD [p−]) decrease by 0.6 times over 7 years. Among laser treatments, laser iridectomy is the most common in FY 2020, which accounts for 56% of all laser treatments, and decreases over 7 years. Gonio photocoagulation increases 2.2 times for 7 years.

**Figure 4:**
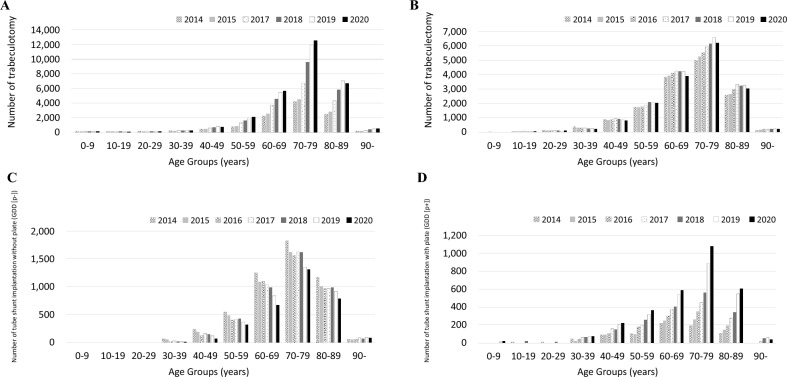
Age-stratified number of common surgical treatments for glaucoma. The age-stratified numbers of trabeculotomy, trabeculectomy, glaucoma drainage device without plate (GDD [p−]), and glaucoma drainage device with plate (GDD [p+]) are presented. Data for trabeculotomy in fiscal year (FY) 2016 are missing due to the regulations of the Ministry of Health, Labour and Welfare. In FY 2020, the number of surgeries in the 70–79-year age group is the highest among all surgical types. In the 70–79-year group, trabeculotomy, trabeculectomy, and GDD (p+) are increased 3.0, 1.2, and 5.7 times, respectively, while GDD (p-) decreased over 7 years.

Figure 2 shows the trend in glaucoma surgeries from FY 2014 to 2020. Figure 2A shows the number of surgical treatments, and Fig. 2B shows the number of laser treatments. The number of surgical treatments was increased 1.8 times for 7 years. Laser treatments remained at approximately 55,000 cases from FY 2014 to 2017, and then increased to approximately 60,000 cases (detailed numbers for each count are shown in Online Supplementary Table 1). Figure 3 and Online Supplementary Fig. 1 show the number of glaucoma surgeries according to surgical type. Among surgical treatments, trabeculotomy was the most common in FY 2020, which accounted for nearly half of all surgical treatments, and 2.6 times higher than that in FY 2014. Despite the overall increase in surgical treatments, the GDD (p−) was decreased by 0.6 times over 7 years. Among laser treatments, laser iridectomy was the most common in FY 2020, which accounted for 56% of all laser treatments, and decreased over 7 years. Gonio photocoagulation was increased 2.2 times over 7 years (detailed numbers for each count are shown in Online Supplementary Table 4). Figure 4 shows the age-stratified number of trabeculotomy, trabeculectomy, GDD (p−), and GDD (p+), which are common surgical treatments for glaucoma. Data for trabeculotomy in FY 2016 were missing because of MHLW regulations. In FY 2020, for all surgical types, the number of surgeries in the 70–79-year age group was the highest; in that age group, trabeculotomy, trabeculectomy, and GDD (p+) were increased by 3.0, 1.2, and 5.7 times, restrictively, while GDD (p−) decreased over 7 years (detailed numbers for each count are shown in Online Supplementary Table 5).

**Figure 5:**
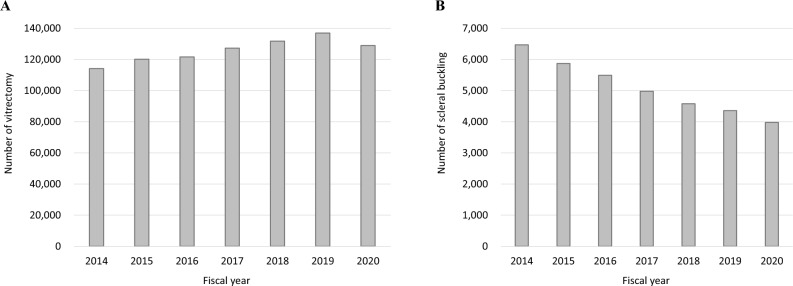
Trend of vitreoretinal surgeries. The number of vitrectomy (**A**) and scleral buckling (**B**) from fiscal year (FY) 2014 to 2020 are presented. The number of vitrectomy is increased 1.2 times from FY 2014 to 2019 but decreased in FY 2020, whereas that of scleral buckling decreased by 0.45 times over 7 years.

**Figure 6:**
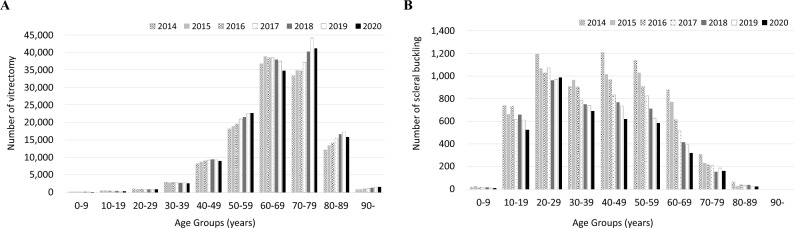
Age-stratified number of vitrectomy and scleral buckling. The peak of vitrectomy shifted from the 60–69-year age group to the 70–79-year age group for 7 years. The number of scleral buckling is decreased in most age groups for 7 years; in particular, the decreases are greater in the 40–49-, 50–59-, and 60–69-year age groups than in the younger age groups.

**Figure 7:**
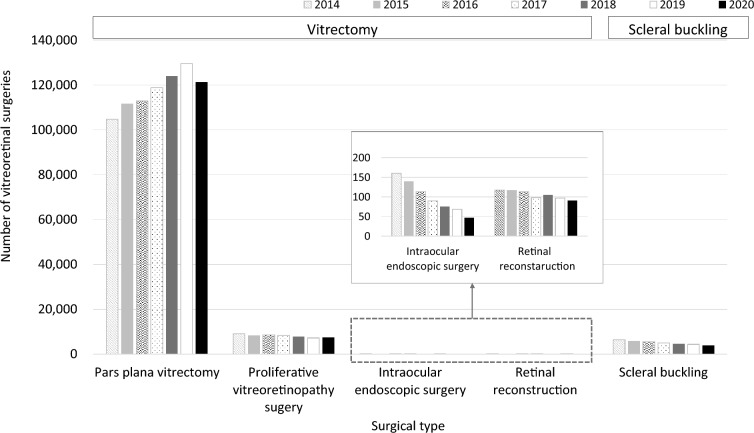
Number of vitreoretinal surgeries by surgical type. Pars plana vitrectomy is increased until fiscal year (FY) 2019 but decreased in FY 2020. Proliferative vitreoretinopathy surgery, endoscopic surgery, retinal reconstruction, and scleral buckling have decreased over 7 years.

Figure 5 shows the trend of vitreoretinal surgeries from FY 2014 to 2020. Figure 5A shows the number of vitrectomy, while Fig. 5B the number of scleral bucklings. The number of vitrectomy was increased 1.2 times from FY 2014 to 2019 but decreased in FY 2020, whereas that of scleral buckling decreased by 0.45 times over 7 years (detailed numbers for each count are shown in Online Supplementary Table 6). Figure 6 shows the age-stratified numbers of vitrectomy and scleral buckling. The peak of vitrectomy shifted from the 60–69-year age group to the 70–79-year age group for 7 years. The number of scleral buckling was decreased in most age groups for 7 years; in particular, the reduction was greater in the 40–49, 50–59, and 60–69 age groups than in the younger age groups (detailed numbers for each count are shown in Online Supplementary Table 7). Figure 7 and Online Supplementary Fig. 2 show the number of vitreoretinal surgeries performed according to the surgical type. The number of pars plana vitrectomy was increased until FY 2019 but decreased until FY 2020. The use of proliferative vitreoretinopathy surgery, endoscopic surgery, retinal reconstruction, and scleral buckling was decreased over the past 7 years (detailed numbers for each count are shown in Online Supplementary Table 6).

## Discussion

Using comprehensive nationwide claims data, this study analyzed trends in ophthalmic surgery in Japan. Cataract surgeries remained stable, whereas glaucoma surgeries and vitrectomies showed distinct patterns. These insights can inform healthcare planning and resource allocation, especially for aging populations. Furthermore, the study emphasizes the potential impact of minimally invasive ophthalmic surgeries on future surgical practices.

The observed ascending trend in ophthalmic surgeries among individuals over 70 years of age aligns with the well-established correlation between aging and age-related eye diseases such as cataracts and glaucoma. This demographic shift urges healthcare systems to meet the increasing demand for surgical interventions targeting these conditions. Furthermore, the rising older population necessitates a proactive approach to educate and train ophthalmic surgeons to effectively address the anticipated surge in the number surgical cases. Not only in Japan, but also in New Zealand, Norway, and Germany, the number of ophthalmic surgeries increased along with the growth of the aging population^12–14^. Countries need to learn from each other how to respond to the increasing demand for ophthalmic surgery and face the common challenges of eye care for the elderly.

The study revealed a trend in the number of surgeries according to the surgical type. In the surgical treatment of glaucoma, the numbers of trabeculotomy and iStent approaches were increased more than that of trabeculectomy. In vitreoretinal surgery, the incidence of pars plana vitrectomy was increased, whereas that of scleral buckling decreased. Such increase in trabeculotomy, iStent, and pars plana vitrectomy may be due to the widespread use of MIGS and MIVS. This shift toward minimally invasive surgery has the potential to expand surgical indications and reduce the risk of postoperative complications. This may lead to improved patient satisfaction and shorter recovery times. MIGS and MIVS will continue to develop, and ophthalmic outcomes are expected to improve. However, the demand for conventional techniques, such as trabeculectomy and scleral buckling, persists; therefore, training in these techniques is important. Trabeculectomy is appropriate for normal-tension glaucoma, which requires lowering the intraocular pressure to approximately 10 mmHg. In Germany and the United States, as well, MIGS increased but trabeculectomy decreased^14,15^. On the other hand, in this study, trabeculectomy has increased along with the increase in MIGS until 2019. This difference may be due to the fact that NTG patients are more common in Asian. The decrease in scleral buckling was smaller in younger patients than in the middle-aged patients. This could be because scleral buckling is safer than pars plana vitrectomy in younger patients. If these conventional techniques become less invasive, the outcomes of ophthalmic surgery should be further improved.

In Japan, the first COVID-19 cases were identified in January 2020^16^, followed by an outbreak on a cruise ship in February^17^. Owing to the spread of the infection, the Japanese government declared a state of emergency regarding COVID-19 on April 7^18^, 2020. Three waves of the infection occurred in 2020. The COVID-19 pandemic has significantly affected healthcare systems and services. Limited medical resources and prioritization of COVID-19 patients have resulted in a postponement of routine general outpatient care and scheduled surgeries. Thus, the COVID-19 pandemic may have affected trends in ophthalmic surgery as well. The current study found that the number of cataract and vitreoretinal surgeries was decreased in 2020 (Figs. 1A and 5), whereas that of glaucoma surgeries did not (Fig. 2). This indicates that cataract and vitreoretinal surgeries were postponed. The reason for the postponement of vitreoretinal surgery may be that in many cases, such as those of surgery of the epiretinal membrane, can be rescheduled. The widespread use of MIVS may have expanded the indications for surgery and even cases that did not require surgery were treated before the COVID-19 pandemic. Previous reports have also showed a decrease in cataract surgeries during the COVID-19 pandemic^19,20^. While glaucoma surgeries did not decrease in Japan, they did in Italy and Brazil^21,22^. This difference may be attributed to variations in the scale of the COVID-19 outbreak within each country. In Italy and Brazil, lockdowns were implemented during the pandemic. Postponement of glaucoma surgery leads to irreversible vision loss, but these social conditions may have forced the postponement of glaucoma surgery.

This study has several limitations that warrant caution when interpreting the results. First, the surgical code defined by the MHLW makes it difficult to evaluate differences between surgical techniques in detail. It is not possible to distinguish between MIGS/MIVS and the conventional methods. For example, trabeculotomy (Medical Practice Code 150088410) includes conventional techniques using the approach from the extraocular side, while MIGS, employs the approach from the intraocular side; however, these cannot be distinguished in the claims issued before March 2022. Tube shunt implantation with a plate (Medical Practice Code 150373010) in glaucoma surgery includes two types, the Ahmed glaucoma valve and the Baerveldt glaucoma implant, which could not be distinguished either. We hope these surgical techniques will be classified in greater detail in future studies. Second, because of the MHLW regulations, rows containing a small number (0–9) are masked; thus, some of the age and sex stratification data are missing. However, its impact on the results was minimal. We expect this rule to be revised in the future. Finally, although the NDB covers medical care for almost all Japanese individuals, medical care not covered by health insurance, such as those provided by social welfare, automobile liability insurance, and workers' compensation insurance, is not included in the NDB^3,5,6,23^. Therefore, the results may have been underestimated. However, because such cases are rare, we believe that their influence is minimal.

In conclusion, the aging population, minimally invasive surgery, and the COVID-19 pandemic may have influenced the number of ophthalmic surgeries in the last 7 years. These findings may be useful for selecting surgical types and allocating medical resources.

### Supplementary Information

Supplementary Figure 1.

Supplementary Figure 2.

Supplementary Table 1.

Supplementary Table 2.

Supplementary Table 3.

Supplementary Table 4.

Supplementary Table 5.

Supplementary Table 6.

Supplementary Table 7.

## Data Availability

The datasets generated during and analyzed during the current study are available from the corresponding author on reasonable request.
