# Supplementary material for: Annual trends of ophthalmic surgeries in Japan’s super-aged society, 2014–2020: a national claims database study
Source: Sci Rep. 2023 Dec 18;13:22884. doi: 10.1038/s41598-023-49705-x (PMC10739960; doi:10.1038/s41598-023-49705-x)

Supplementary Figure 1. The number of glaucoma surgeries by surgical type

The number of surgical treatments (A) and laser treatments (B) are presented.

Among surgical treatments, trabeculotomy was the most common in the fiscal year (FY) 2020, which accounted for nearly half of all surgical treatments, and was 2.6 times higher than that in FY 2014. Despite an overall increase of surgical treatments, glaucoma drainage device without plate (GDD [p-]) decreased 0.6 times over 7 years. Among laser treatments, laser iridectomy was the most common in FY 2020, which accounted for 56% of all laser treatments, and decreased over 7 years. Gonio photocoagulation increased 2.2 times over 7 years.

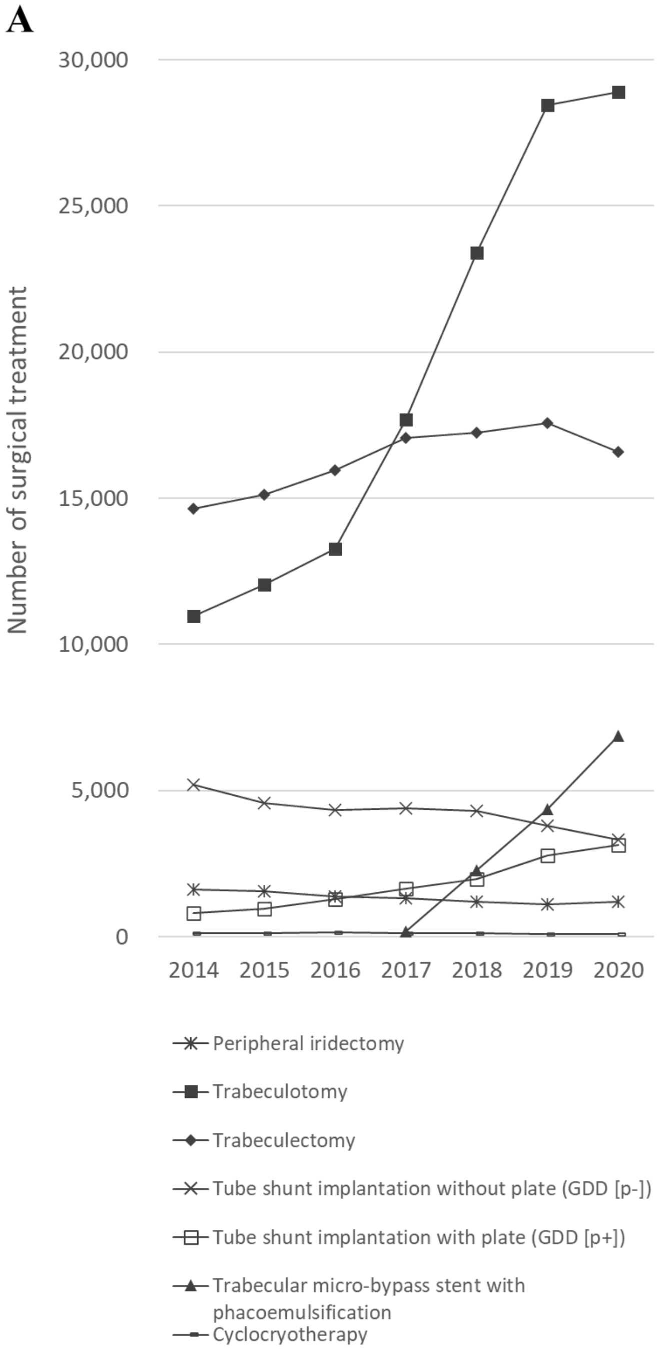

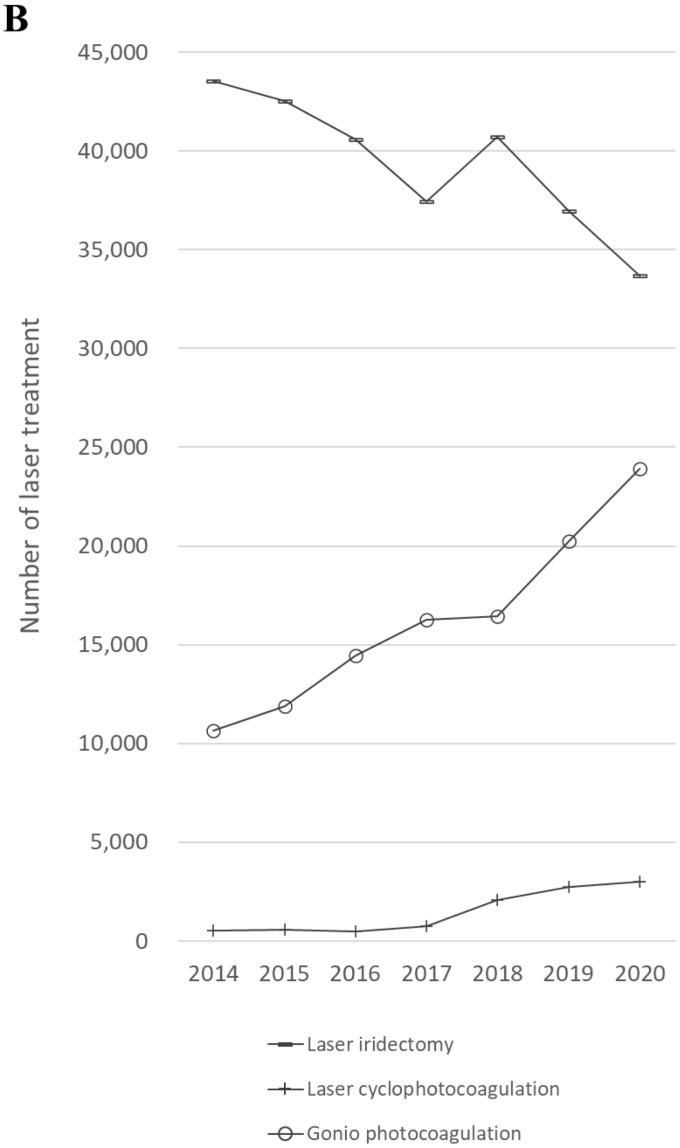

Supplement: Supplementary file 1 — Supplementary Figure 1. [file 41598_2023_49705_MOESM1_ESM.pdf]
