# Supplementary material for: Annual trends of ophthalmic surgeries in Japan’s super-aged society, 2014–2020: a national claims database study
Source: Sci Rep. 2023 Dec 18;13:22884. doi: 10.1038/s41598-023-49705-x (PMC10739960; doi:10.1038/s41598-023-49705-x)

Supplementary Figure 2. The number of vitreoretinal surgeries by surgical type

Pars plana vitrectomy increased until the fiscal year (FY) 2019 but decreased until FY 2020. Proliferative vitreoretinopathy surgery, endoscopic surgery, retinal reconstruction, and scleral buckling has decreased over the past 7 years.

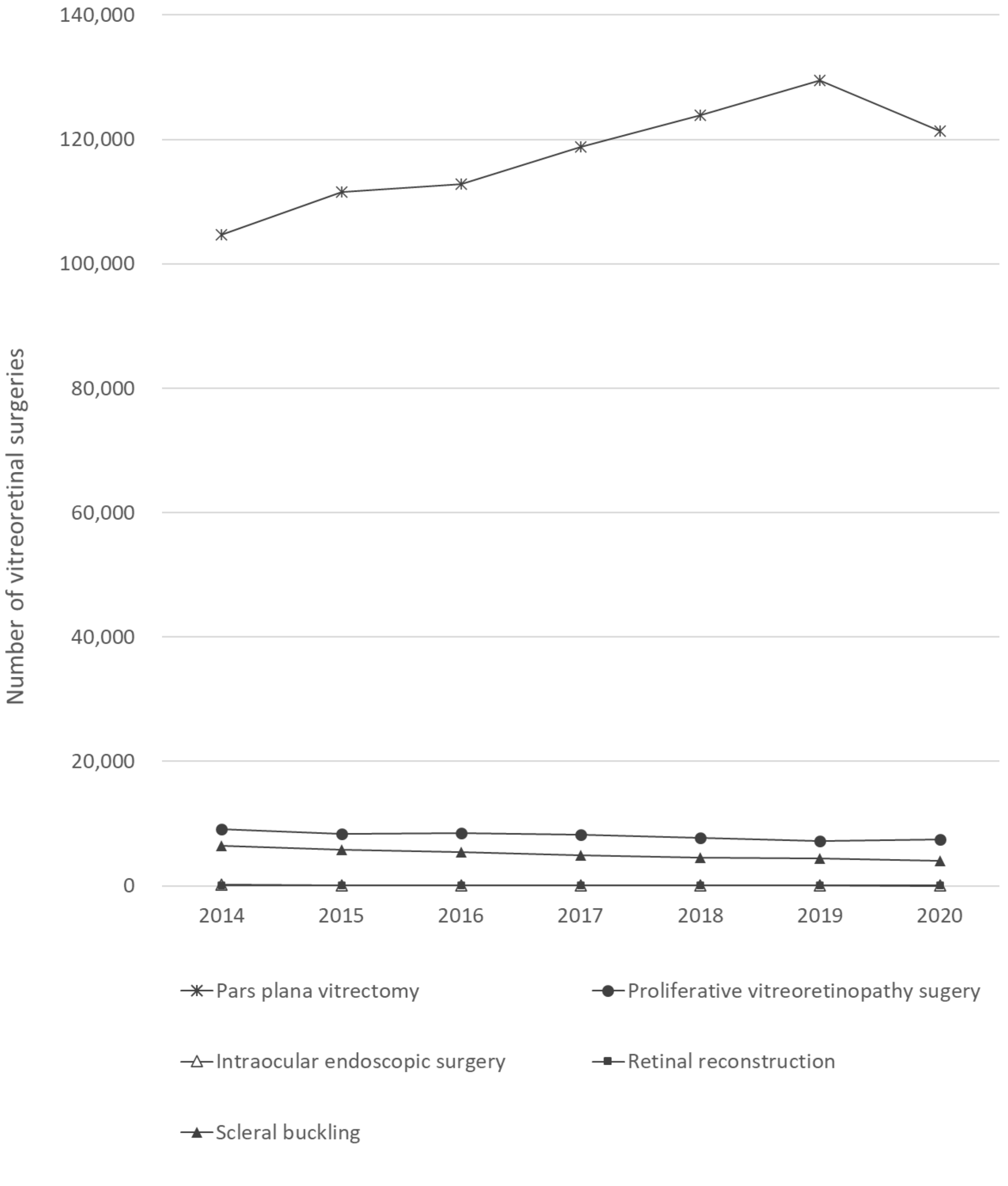

Supplement: Supplementary file 2 — Supplementary Figure 2. [file 41598_2023_49705_MOESM2_ESM.pdf]
