# Supplementary material for: Annual trends of ophthalmic surgeries in Japan’s super-aged society, 2014–2020: a national claims database study
Source: Sci Rep. 2023 Dec 18;13:22884. doi: 10.1038/s41598-023-49705-x (PMC10739960; doi:10.1038/s41598-023-49705-x)
Supplement: Supplementary file 3 — Supplementary Table 1. [file 41598_2023_49705_MOESM3_ESM.docx]

| **Supplementary Table 1. Japanese surgical codes and medical practice codes used to identify cataract surgery, glaucoma surgery, and vitreoretinal surgery.** | | | | | |
| --- | --- | --- | --- | --- | --- |
|  | Surgical code |  | Medical practice code |  | Surgical type |
| Cataract surgery | K282 |  | 150253010 |  | Lens reconstruction (With intraocular lens) (Other) |
|  |  |  | 150315610 |  | Lens reconstruction (Without intraocular lens) |
|  |  |  | 150356210 |  | Lens reconstruction (With intraocular lens) (Suture fixation of intraocular lens) |
|  |  |  | 150356310 |  | Lens reconstruction (Planned posterior capsule incision) |
|  |  |  | 150380950 |  | Lens reconstruction (With capsular tension ring and suture fixation) |
|  |  |  | 150381050 |  | Lens reconstruction (Without capsular tension ring and suture fixation) |
|  | A400 |  | 190179210 |  | Basic fee 3 for short-stay surgery (Lens reconstruction, With intraocular lens, Other, One eye) |
|  |  |  | 190179310 |  | Basic fee 3 for short-stay surgery (Lens reconstruction, With intraocular lens, Other, One eye) (medical treatment) |
|  |  |  | 190179410 |  | Basic fee 3 for short-stay surgery (Lens reconstruction, Without intraocular lens, One eye) |
|  |  |  | 190179510 |  | Basic fee 3 for short-stay surgery (Lens reconstruction, Without intraocular lens, One eyes) (medical treatment) |
|  |  |  | 190182750 |  | Basic fee 3 for short-stay surgery (Lens reconstruction, Without capsular tension ring and suture fixation) |
|  |  |  | 190182850 |  | Basic fee 3 for short-stay surgery (Lens reconstruction, Without capsular tension ring and suture fixation) (medical treatment) |
|  |  |  | 190195910 |  | Basic fee 3 for short-stay surgery (Lens reconstruction, With intraocular lens, Other, Both eyes) |
|  |  |  | 190196010 |  | Basic fee 3 for short-stay surgery (Lens reconstruction, With intraocular lens, Other, Both eyes) (medical treatment) |
|  |  |  | 190196110 |  | Basic fee 3 for short-stay surgery (Lens reconstruction, Without intraocular lens, Both eyes) |
|  |  |  | 190196210 |  | Basic fee 3 for short-stay surgery (Lens reconstruction, Without intraocular lens, Both eyes) (medical treatment) |
| Glaucoma surgery | K268 |  | 150087510 |  | Peripheral iridectomy |
|  |  |  | 150088410 |  | Trabeculotomy |
|  |  |  | 150335910 |  | Trabeculectomy |
|  |  |  | 150356010 |  | Tube shunt implantation without plate |
|  |  |  | 150373010 |  | Tube shunt implantation with plate |
|  |  |  | 150395150 |  | Trabecular micro-bypass stent with phacoemulsification |
|  | K270 |  | 150088710 |  | Laser iridectomy |
|  | K271 |  | 150088810 |  | Laser cyclophotocoagulation |
|  | K272 |  | 150088910 |  | Cyclocryotherapy |
|  | K273 |  | 150089010 |  | Gonio photocoagulation |
| Vitreoretinal surgery | K275 |  | 150089410 |  | Scleral buckling |
|  | K280 |  | 150090610 |  | Pars plana vitrectomy (Other) |
|  |  |  | 150274010 |  | Pars plana vitrectomy (Including retinal adherent tissue) |
|  | K280-2 |  | 150356110 |  | Intraocular endoscopic surgery |
|  | K281 |  | 150252810 |  | Proliferative vitreoretinopathy surgery |
|  | K281-2 |  | 150373110 |  | Retinal reconstruction |
