# Supplementary material for: Annual trends of ophthalmic surgeries in Japan’s super-aged society, 2014–2020: a national claims database study
Source: Sci Rep. 2023 Dec 18;13:22884. doi: 10.1038/s41598-023-49705-x (PMC10739960; doi:10.1038/s41598-023-49705-x)
Supplement: Supplementary file 4 — Supplementary Table 2. [file 41598_2023_49705_MOESM4_ESM.docx]

| **Supplementary Table 2. The total number of cataract surgeries, glaucoma surgeries, and vitreoretinal surgeries from fiscal year 2014 to 2020.** | | | | | | | | |  |
| --- | --- | --- | --- | --- | --- | --- | --- | --- | --- |
|  |  |  |  |  |  |  |  |  |  |
| Fiscal year |  | Cataract surgery |  | Glaucoma surgery | |  | Vitreoretinal surgery |  |  |
|  |  |  |  | Surgical treatment | Laser treatment |  |  |  |  |
| 2014 |  | 1,415,267 |  | 33,340 | 54,679 |  | 120,530 |  |  |
| 2015 |  | 1,472,939 |  | 34,340 | 54,963 |  | 125,963 |  |  |
| 2016 |  | 1,457,399 |  | 36,324 | 55,499 |  | 127,086 |  |  |
| 2017 |  | 1,485,167 |  | 42,390 | 54,450 |  | 132,194 |  |  |
| 2018 |  | 1,483,105 |  | 50,497 | 59,238 |  | 136,287 |  |  |
| 2019 |  | 1,572,057 |  | 58,143 | 59,941 |  | 141,226 |  |  |
| 2020 |  | 1,448,997 |  | 60,108 | 60,547 |  | 132,879 |  |  |
