# Supplementary material for: Annual trends of ophthalmic surgeries in Japan’s super-aged society, 2014–2020: a national claims database study
Source: Sci Rep. 2023 Dec 18;13:22884. doi: 10.1038/s41598-023-49705-x (PMC10739960; doi:10.1038/s41598-023-49705-x)
Supplement: Supplementary file 5 — Supplementary Table 3. [file 41598_2023_49705_MOESM5_ESM.docx]

| **Supplementary Table 3. The number of cataract surgeries by age group from fiscal year 2014 to 2020.** | | | | | | | | | | |
| --- | --- | --- | --- | --- | --- | --- | --- | --- | --- | --- |
| Fiscal year | Age group | | | | | | | | | |
|  | 0-9 | 10-19 | 20-29 | 30-39 | 40-49 | 50-59 | 60-69 | 70-79 | 80-89 | 90- |
| 2014 | 438 | 638 | 1,809 | 4,970 | 18,821 | 67,598 | 309,737 | 619,668 | 364,501 | 26,807 |
| 2015 | 417 | 484 | 1,301 | 3,669 | 13,439 | 50,299 | 256,447 | 533,638 | 321,928 | 24,373 |
| 2016 | 314 | 418 | 1,217 | 3,540 | 14,281 | 50,433 | 260,916 | 528,226 | 326,309 | 24,937 |
| 2017 | 368 | 290 | 1,085 | 3,420 | 15,773 | 55,716 | 239,351 | 479,074 | 268,594 | 18,625 |
| 2018 | 458 | 511 | 1,446 | 4,291 | 19,841 | 69,868 | 288,513 | 670,576 | 395,381 | 32,087 |
| 2019 | 418 | 577 | 1,477 | 4,229 | 19,799 | 73,561 | 289,189 | 732,692 | 414,981 | 34,983 |
| 2020 | 456 | 564 | 1,586 | 4,545 | 21,368 | 79,253 | 270,810 | 688,302 | 354,168 | 27,801 |
