# Supplementary material for: Annual trends of ophthalmic surgeries in Japan’s super-aged society, 2014–2020: a national claims database study
Source: Sci Rep. 2023 Dec 18;13:22884. doi: 10.1038/s41598-023-49705-x (PMC10739960; doi:10.1038/s41598-023-49705-x)
Supplement: Supplementary file 6 — Supplementary Table 4. [file 41598_2023_49705_MOESM6_ESM.docx]

| **Supplementary Table 4 The number of glaucoma surgeries by surgery type from fiscal year 2014 to 2020.** | | | | | | | | | | | | | | |
| --- | --- | --- | --- | --- | --- | --- | --- | --- | --- | --- | --- | --- | --- | --- |
| Surgery type |  | Fiscal year | | | | | | | | | | | | |
|  |  | 2014 |  | 2015 |  | 2016 |  | 2017 |  | 2018 |  | 2019 |  | 2020 |
| Surgical treatment |  |  |  |  |  |  |  |  |  |  |  |  |  |  |
| Peripheral iridectomy |  | 1,608 |  | 1,543 |  | 1,367 |  | 1,310 |  | 1,194 |  | 1,100 |  | 1,209 |
| Trabeculotomy |  | 10,957 |  | 12,045 |  | 13,260 |  | 17,692 |  | 23,394 |  | 28,435 |  | 28,900 |
| Trabeculectomy |  | 14,644 |  | 15,114 |  | 15,957 |  | 17,063 |  | 17,235 |  | 17,576 |  | 16,582 |
| Tube shunt implantation without plate (GDD [p-]) |  | 5,200 |  | 4,562 |  | 4,321 |  | 4,388 |  | 4,318 |  | 3,789 |  | 3,327 |
| Tube shunt implantation with plate (GDD [p+]) |  | 817 |  | 954 |  | 1,279 |  | 1,640 |  | 1,980 |  | 2,773 |  | 3,129 |
| Trabecular micro-bypass stent with phacoemulsification |  | 0 |  | 0 |  | 0 |  | 170 |  | 2,261 |  | 4,370 |  | 6,859 |
| Cyclocryotherapy |  | 114 |  | 122 |  | 140 |  | 127 |  | 115 |  | 100 |  | 102 |
| Laser treatment |  |  |  |  |  |  |  |  |  |  |  |  |  |  |
| Laser iridectomy |  | 43,518 |  | 42,504 |  | 40,583 |  | 37,447 |  | 40,707 |  | 36,951 |  | 33,661 |
| Laser cyclophotocoagulation |  | 517 |  | 567 |  | 482 |  | 744 |  | 2,072 |  | 2,751 |  | 2,983 |
| Gonio photocoagulation |  | 10,644 |  | 11,892 |  | 14,434 |  | 16,259 |  | 16,459 |  | 20,239 |  | 23,903 |
