# Supplementary material for: Annual trends of ophthalmic surgeries in Japan’s super-aged society, 2014–2020: a national claims database study
Source: Sci Rep. 2023 Dec 18;13:22884. doi: 10.1038/s41598-023-49705-x (PMC10739960; doi:10.1038/s41598-023-49705-x)
Supplement: Supplementary file 7 — Supplementary Table 5. [file 41598_2023_49705_MOESM7_ESM.docx]

| **Supplementary Table 5 The number of trabeculotomy, trabeculectomy, tube shunt implantation without plate (GDD [p-]) and tube shunt implantation with plate (GDD [p+]) by age group from fiscal year 2014 to 2020.** | | | | | | | | | | |  |
| --- | --- | --- | --- | --- | --- | --- | --- | --- | --- | --- | --- |
|  |  |  |  |  |  |  |  |  |  |  |  |
|  |  |  |  |  |  |  |  |  |  |  |  |
| **Trabeculotomy** |  |  |  |  |  |  |  |  |  |  |  |
| Fiscal year | Age group | | | | | | | | | |  |
|  | 0-9 | 10-19 | 20-29 | 30-39 | 40-49 | 50-59 | 60-69 | 70-79 | 80-89 | 90- |  |
| 2014 | 171 | 101 | 153 | 233 | 443 | 757 | 2,210 | 4,244 | 2,478 | 167 |  |
| 2015 | 149 | 125 | 124 | 207 | 482 | 851 | 2,540 | 4,509 | 2,821 | 201 |  |
| 2016 | 0 | 0 | 0 | 0 | 72 | 160 | 590 | 1,496 | 942 | 56 |  |
| 2017 | 135 | 110 | 174 | 266 | 605 | 1,302 | 3,741 | 6,734 | 4,315 | 275 |  |
| 2018 | 144 | 116 | 144 | 275 | 691 | 1,613 | 4,560 | 9,565 | 5,824 | 442 |  |
| 2019 | 146 | 116 | 176 | 276 | 788 | 1,984 | 5,446 | 11,935 | 7,056 | 481 |  |
| 2020 | 125 | 86 | 133 | 261 | 730 | 2,105 | 5,656 | 12,526 | 6,702 | 509 |  |
|  |  |  |  |  |  |  |  |  |  |  |  |
|  |  |  |  |  |  |  |  |  |  |  |  |
| **Trabeculectomy** | | | | | | | | | | |  |
| Fiscal year | Age group | | | | | | | | | |  |
|  | 0-9 | 10-19 | 20-29 | 30-39 | 40-49 | 50-59 | 60-69 | 70-79 | 80-89 | 90- |  |
| 2014 | 0 | 28 | 112 | 367 | 857 | 1,746 | 3,803 | 4,992 | 2,576 | 132 |  |
| 2015 | 0 | 43 | 112 | 316 | 837 | 1,730 | 3,912 | 5,271 | 2,652 | 177 |  |
| 2016 | 12 | 26 | 92 | 284 | 865 | 1,781 | 4,116 | 5,530 | 2,987 | 196 |  |
| 2017 | 0 | 51 | 119 | 284 | 950 | 1,891 | 4,252 | 5,953 | 3,323 | 199 |  |
| 2018 | 0 | 26 | 98 | 255 | 901 | 2,084 | 4,224 | 6,147 | 3,232 | 213 |  |
| 2019 | 0 | 26 | 64 | 252 | 851 | 2,049 | 4,207 | 6,589 | 3,260 | 226 |  |
| 2020 | 0 | 42 | 93 | 228 | 803 | 2,036 | 3,880 | 6,200 | 3,026 | 224 |  |
|  |  |  |  |  |  |  |  |  |  |  |  |
|  |  |  |  |  |  |  |  |  |  |  |  |
| **Tube shunt implantation without plate (GDD [p-])** | | | | | | | | | | |  |
| Fiscal year | Age group | | | | | | | | | |  |
|  | 0-9 | 10-19 | 20-29 | 30-39 | 40-49 | 50-59 | 60-69 | 70-79 | 80-89 | 90- |  |
| 2014 | 0 | 0 | 0 | 69 | 240 | 548 | 1,248 | 1,828 | 1,176 | 63 |  |
| 2015 | 0 | 0 | 0 | 57 | 194 | 484 | 1,090 | 1,618 | 1,010 | 60 |  |
| 2016 | 0 | 0 | 0 | 24 | 127 | 404 | 1,100 | 1,563 | 968 | 64 |  |
| 2017 | 0 | 0 | 0 | 32 | 168 | 413 | 1,037 | 1,629 | 970 | 90 |  |
| 2018 | 0 | 0 | 0 | 20 | 148 | 430 | 988 | 1,622 | 989 | 75 |  |
| 2019 | 0 | 0 | 0 | 23 | 122 | 374 | 838 | 1,356 | 921 | 95 |  |
| 2020 | 0 | 0 | 0 | 14 | 72 | 325 | 674 | 1,310 | 791 | 88 |  |
|  |  |  |  |  |  |  |  |  |  |  |  |
|  |  |  |  |  |  |  |  |  |  |  |  |
| **Tube shunt implantation with plate (GDD [p+])** | | | | | | | | | | |  |
| Fiscal year | Age group | | | | | | | | | |  |
|  | 0-9 | 10-19 | 20-29 | 30-39 | 40-49 | 50-59 | 60-69 | 70-79 | 80-89 | 90- |  |
| 2014 | 0 | 10 | 10 | 44 | 89 | 104 | 218 | 190 | 110 | 0 |  |
| 2015 | 0 | 0 | 0 | 20 | 93 | 95 | 246 | 263 | 147 | 0 |  |
| 2016 | 0 | 0 | 0 | 40 | 106 | 176 | 299 | 349 | 192 | 0 |  |
| 2017 | 0 | 0 | 0 | 62 | 160 | 198 | 376 | 450 | 276 | 16 |  |
| 2018 | 0 | 22 | 13 | 65 | 151 | 259 | 407 | 559 | 341 | 53 |  |
| 2019 | 11 | 0 | 0 | 72 | 206 | 318 | 538 | 885 | 548 | 60 |  |
| 2020 | 22 | 0 | 0 | 74 | 224 | 366 | 586 | 1,080 | 605 | 38 |  |
