# Supplementary material for: Annual trends of ophthalmic surgeries in Japan’s super-aged society, 2014–2020: a national claims database study
Source: Sci Rep. 2023 Dec 18;13:22884. doi: 10.1038/s41598-023-49705-x (PMC10739960; doi:10.1038/s41598-023-49705-x)
Supplement: Supplementary file 8 — Supplementary Table 6. [file 41598_2023_49705_MOESM8_ESM.docx]

| **Supplementary Table 6 The number of vitreoretinal surgeries by surgery type from fiscal year 2014 to 2020.** | | | | | | | | | | | | | | |
| --- | --- | --- | --- | --- | --- | --- | --- | --- | --- | --- | --- | --- | --- | --- |
| Surgery type |  | Fiscal year | | | | | | | | | | |  |  |
|  |  | 2014 |  | 2015 |  | 2016 |  | 2017 |  | 2018 |  | 2019 |  | 2020 |
| Vitrectomy |  | 114,061 |  | 120,094 |  | 121,596 |  | 127,215 |  | 131,712 |  | 136,871 |  | 128,904 |
| Pars plane vitrectomy |  | 104,618 |  | 111,542 |  | 112,824 |  | 118,737 |  | 123,838 |  | 129,519 |  | 121,301 |
| Intraocular endoscopic surgery |  | 160 |  | 140 |  | 114 |  | 90 |  | 75 |  | 69 |  | 47 |
| Proliferative vitreoretinopathy surgery |  | 9,166 |  | 8,295 |  | 8,545 |  | 8,290 |  | 7,694 |  | 7,186 |  | 7,465 |
| Retinal reconstruction |  | 117 |  | 117 |  | 113 |  | 98 |  | 105 |  | 97 |  | 91 |
| Scleral buckling |  | 6,469 |  | 5,869 |  | 5,490 |  | 4,979 |  | 4,575 |  | 4,355 |  | 2,924 |
