# Supplementary material for: Annual trends of ophthalmic surgeries in Japan’s super-aged society, 2014–2020: a national claims database study
Source: Sci Rep. 2023 Dec 18;13:22884. doi: 10.1038/s41598-023-49705-x (PMC10739960; doi:10.1038/s41598-023-49705-x)
Supplement: Supplementary file 9 — Supplementary Table 7. [file 41598_2023_49705_MOESM9_ESM.docx]

| **Supplementary Table 7 The number of vitrectomy and scleral buckling by age group from fiscal year 2014 to 2020.** | | | | | | | | | | |
| --- | --- | --- | --- | --- | --- | --- | --- | --- | --- | --- |
|  |  |  |  |  |  |  |  |  |  |  |
| **Vitrectomy** | | | | | | | | | | |
| Fiscal year | Age group | | | | | | | | | |
|  | 0-9 | 10-19 | 20-29 | 30-39 | 40-49 | 50-59 | 60-69 | 70-79 | 80-89 | 90- |
| 2014 | 113 | 490 | 1,003 | 2,916 | 8,172 | 18,147 | 36,774 | 33,321 | 12,127 | 816 |
| 2015 | 157 | 449 | 924 | 2,767 | 8,692 | 18,834 | 38,799 | 34,811 | 13,421 | 890 |
| 2016 | 148 | 435 | 901 | 2,828 | 9,098 | 19,497 | 38,401 | 34,703 | 14,133 | 1,101 |
| 2017 | 158 | 385 | 868 | 2,812 | 9,240 | 20,937 | 38,631 | 37,191 | 15,446 | 1,165 |
| 2018 | 107 | 374 | 908 | 2,753 | 9,449 | 21,564 | 37,967 | 40,278 | 16,570 | 1,341 |
| 2019 | 96 | 368 | 860 | 2,763 | 9,308 | 22,660 | 37,490 | 44,122 | 17,259 | 1,555 |
| 2020 | 62 | 324 | 910 | 2,536 | 8,986 | 22,637 | 34,744 | 41,152 | 15,773 | 1,546 |
|  |  |  |  |  |  |  |  |  |  |  |
|  |  |  |  |  |  |  |  |  |  |  |
| **Scleral buckling** | | | | | | | | | | |
| Fiscal year | Age group | | | | | | | | | |
|  | 0-9 | 10-19 | 20-29 | 30-39 | 40-49 | 50-59 | 60-69 | 70-79 | 80-89 | 90- |
| 2014 | 20 | 736 | 1,191 | 908 | 1,204 | 1,141 | 879 | 307 | 63 | 0 |
| 2015 | 26 | 661 | 1,065 | 965 | 1,012 | 1,028 | 770 | 233 | 29 | 0 |
| 2016 | 20 | 732 | 1,027 | 905 | 964 | 907 | 613 | 216 | 40 | 0 |
| 2017 | 16 | 618 | 1,074 | 785 | 831 | 825 | 521 | 211 | 36 | 0 |
| 2018 | 17 | 659 | 962 | 750 | 769 | 713 | 416 | 152 | 37 | 0 |
| 2019 | 10 | 611 | 968 | 739 | 735 | 629 | 392 | 188 | 26 | 0 |
| 2020 | 11 | 524 | 986 | 691 | 619 | 582 | 321 | 162 | 23 | 0 |
